# Metabolic Engineering of *Escherichia coli* for Enhanced Production of Naringenin 7-Sulfate and Its Biological Activities

**DOI:** 10.3389/fmicb.2018.01671

**Published:** 2018-07-27

**Authors:** Luan L. Chu, Dipesh Dhakal, Hee J. Shin, Hye J. Jung, Tokutaro Yamaguchi, Jae K. Sohng

**Affiliations:** ^1^Department of Life Science and Biochemical Engineering, Sun Moon University, Asan, South Korea; ^2^Department of BT Convergence Pharmaceutical Engineering, Sun Moon University, Asan, South Korea

**Keywords:** sulfotransferase, CRISPRi, *E. coli*, PAPS, metabolic engineering

## Abstract

Flavonoids are one of the predominant groups of plant polyphenols, and these compounds have significant effects on human health and nutrition. Sulfated flavonoids have more favorable attributes compared to their parent compounds such as increased solubility, stability, and bioavailability. In this research, we developed a microbial system to produce sulfated naringenin using *Escherichia coli* expressing a sulfotransferase (ST) from *Arabidopsis thaliana* (At2g03770). This wild-type strain was used as a model system for testing clustered regularly interspaced short palindromic repeats (CRISPR) interference (CRISPRi) metabolic engineering strategies. Using synthetic sgRNA to mediate transcriptional repression of *cysH*, a gene encoding 3′-phosphoadenosine-5′-phosphosulfate (PAPS) ST, which is involved in sulfur metabolism, resulted in an increase in intracellular PAPS accumulation by over 3.28-fold without impairing cell growth. Moreover, naringenin 7-sulfate production by engineering *E. coli* with its *cysH* gene repressed in the open reading frame through CRISPRi was enhanced by 2.83-fold in compared with the wild-type control. To improve the efficiency of biotransformation, the concentration of $SO_{4}^{2-}$, glucose, and substrate were optimized. The bioproductivity of naringenin 7-sulfate was 135.49 μM [∼143.1 mg (47.7 mg L^-1^)] in a 3-L fermenter at 36 h. These results demonstrated that the CRISPRi system was successfully applied for the first time in *E. coli* to develop an efficient microbial strain for production of a sulfated flavonoid. In addition, antibacterial and anticancer activities of naringenin 7-sulfate were investigated and found to be higher than the parent compound.

## Introduction

Flavonoids are major natural phenolic compounds found in plants (Buer et al., 2010). One of the most predominant citrus flavonoids is naringenin and it was found to exhibit various biological effects on human health and nutrition. Naringenin showed anti-estrogenic, antioxidant (Bugianesi et al., 2002), anti-obesity and anti-diabetic activities (Hossain et al., 2016). It has been demonstrated to control non-alcoholic steatohepatitis and associated inflammation (Jadeja and Devkar, 2014). Like flavonoids, most of the naringenin in nature accumulates in a glycosylated form (Gattuso et al., 2007), which improves solubility, storage, and stability of the parent compounds (De Bruyn et al., 2015). In addition to glycosylation, many the sulfate conjugate of flavonoids discovered in the different type of plants (Barron et al., 1998). However, the biological and physiological properties of sulfated flavonoids have not been studied (Totta et al., 2005; Wang et al., 2014).

Sulfation plays important roles in detoxification of xenobiotics (Yi et al., 2006; Chen et al., 2015) and regulating the activity of animal hormones (Geyer et al., 2017). Enzymatic sulfation is catalyzed by a family of sulfotransferases (STs) that transfer the sulfonate group of 3′-phosphoadenosine-5′-phosphosulfate (PAPS) to a hydroxyl group or amino group of acceptor compounds with the parallel formation of 3′-phosphoadenosine-5′-phosphate (PAP) (Paul et al., 2012). Although the sequences of many STs have been deposited to various genome databases of plant (Gidda and Varin, 2006; Marsolais et al., 2007), bacteria (Hossain et al., 2011), and mammalian (Wong et al., 2010; Shimohira et al., 2017), the enzymatic and microbial synthesis of sulfonated natural products still are limited. One of the major problems of *in vitro* sulfation reactions is the use of the costly and unstable sulfate donor, PAPS, which impedes industrial scale-up of the process. Moreover, while chemical synthesis of sulfated compounds is tedious and time-consuming in terms of involving multiple steps (Zhang et al., 2012), PAPS has a poor availability in the cytosol of microbial cells like *Escherichia coli* and *Streptomyces* for production or modification of natural compounds (Sekowska et al., 2000; Nishikawa et al., 2017). Recently, genetic engineering has been used for production or post-modification of the natural compound *via* improving the availability of the common precursor in the biosynthetic pathway. For example, metabolic engineering of *E. coli* in the superpathway of methionine and *S*-adenosyl-L-methionine (SAM) biosynthesis, lead to improved SAM availability, and finally increased anthocyanin *O*-methylation (Cress et al., 2017). The sugar pathway was overexpressed to improve the cytoplasmic pool of NDP-sugars and subsequently expressed glycosyltransferase was used for the biosynthesis of glycosylated flavonoids in *E. coli* (Kim et al., 2015; Pandey et al., 2016). However, the same approach has not been applied to the biosynthesis of sulfated flavonoids.

Genetic editing using the clustered regularly interspaced short palindromic repeat (CRISPR) system has been widely used in diverse organisms including bacteria (Huang et al., 2015; Wu et al., 2015), fungal kingdoms (Wang et al., 2017), plant (Khan et al., 2017), animals (Gandhi et al., 2017), and human cell lines (Bertomeu et al., 2017). In the type II CRISPR, an RNA-guide DNA endonuclease (Cas9) is targeted to specific DNA sequences through a chimeric guide RNA (gRNA), which is a fusion between a precursor CRISPR RNAs (crRNA) and trans-activating CRISPR RNAs (tracrRNAs) (Huang et al., 2015). This gRNA is capable of recognizing sequences target sites for marker-free integration or gene disruption that are followed by the protospacer-adjacent motif (PAM) sequence NGG, N being any nucleotide (Cress et al., 2015). Recently, CRISPR interference (CRISPRi) has been developed which contains a mutation in the Cas9 protein (D10A and H840A) resulted in a lack of endonuclease activity (dCas9), but DNA binding capability remained for inhibition of transcription (Bikard et al., 2013). CRISPRi has been applied to down-regulation of genes in certain pathways *via* metabolic engineering of *E. coli* for production of value natural compounds (Wu et al., 2015; Liang et al., 2016; Gao et al., 2017). However, it has not been reported to regulate a gene in *E. coli* sulfur metabolism.

**FIGURE 1:**
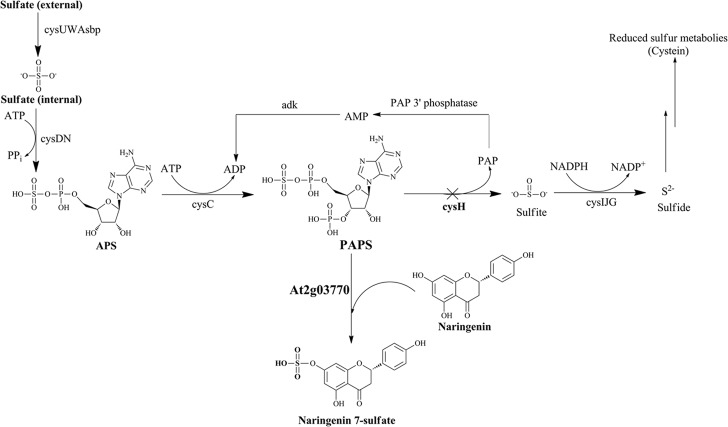
Schematic of sulfur metabolic pathway with strategies for biosynthesis of naringenin 7-sulfate in *E. coli*. *Cross* show the repressed gene in this pathway by using CRISPRi system. Operon *cysUWAsbp*, a sulfate permease; operon *cysDN*, a ATP sulfurylase; *cysC*, APS kinase; *cysH*, PAPS sulfotransferase; operon *cysIJG*, sulfite reductase; *adk*, adenylate kinase; *APS*, adenosine 5′-phosphosulfate; *PAP*, 3′-phosphoadenosine-5′-phosphate.

In this study, we expressed a ST from *Arabidopsis thaliana* (At2g03770) for biosynthesis of sulfated naringenin in *E. coli* BL21 (DE3). Naringenin has been shown to be substrate specificity of At2g03770 (Hashiguchi et al., 2013). Furthermore, we employed a CRISPRi system as a tool for improving PAPS availability by knockdown PAPS ST (*cysH*) in the sulfur metabolism (**Figure 1**), resulted in enhancement the final products. This is the first research that a metabolic engineering approach for conjugating a sulfate moiety generated in the cytoplasm of *E. coli*. The biosynthesized compound was also tested for its potential bioactivities against various pathogens and cancer cell lines.

## Materials and Methods

### Chemicals and Reagents

Restriction enzymes, shrimp alkaline phosphatase, T_4_ polynucleotide kinase, and T_4_ DNA ligase were obtained from New England Biolabs (Hertfordshire, United Kingdom). Standard naringenin, adenosine 3′-phosphate 5′-phosphosulfate lithium salt hydrate (PAPS), adenosine 5′-triphosphate disodium salt hydrate (ATP), adenosine 5′-diphosphate (ADP), deuterium oxide (D_2_O), and dimethyl sulfoxide-*d_6_* (DMSO-*d_6_*) were purchased from Sigma-Aldrich (St. Louis, MO, United States). Isopropyl-β-D-thiogalactopyranoside (IPTG) was obtained from GeneChem Inc. (Daejeon, South Korea). All other chemicals were of the highest grade commercially available.

### Plasmid and Strains Constructions

All the *E. coli* strains and plasmids used in this study are supported in **Table 1**. PCR primers utilized for gene amplification and cloning were synthesized by GenoTech Corp. (Daejeon, South Korea). At2g03770 were codon-optimized for *E. coli*, synthesized, cloned into the plasmid vector pUC57 by General Biosystems, Inc. (NC, United States). The *Bam*HI and *Xho*I sites were added to the 5′ and 3′ ends of the gene. The synthesized gene was restricted by *Bam*HI and *Xho*I digestion, subcloned into the expression vector pET28a (+) to create the pET28a(+)-At2g03770. Then, the recombinant plasmid was transformed into *E. coli* BL21 (DE3) to form the strain used for production (wild-type) (**Table 1**).

**Table 1:** Strains and plasmids used in this study.

Strain/plasmids	Properties/genotype	Source/reference
**Strains**		
*E. coli DH5α*	F-Φ80lacZΔM15 Δ(lacZYA-argF) U169 recA1 endA1 hsdR17 (rK-, mK+) phoA supE44 λ- thi-1 gyrA96 relA1	Novagen
*E. coli* BL21(DE3)	ompT hsdT hsdS (rB^-^ mB^-^) gal (DE3)	Novagen
Wild-type	*E. coli* BL21 (DE3) carrying pET 28a(+)-At2g03770	This study
S1	*E. coli* BL21 (DE3) carrying pET 28a(+)-At2g03770 and pCRISPathBrick-1	This study
S2	*E. coli* BL21 (DE3) carrying pET 28a(+)-At2g03770 and pCRISPathBrick-2	This study
Plasmid vectors		
pET 28a(+)	Expression vector with T7 promoter, p15A ori, Km^r^	Novagen
pET 28a(+)-At3g45070	pET 28a(+) carrying At2g03770 from *A. thaliana*	This study
pCRISPathBrick	pACYC184 (Cm^r^), p15A ori, *Streptococcus pyogenes* dCas9 (D10A, H840A), tracrRNA, non-targeting CRISPR spacer with *Bsa*I site	Cress et al., 2015
CRISPRi-1	pCRISPathBrick, CRISPR spacer targeting *cysH* near promoter	This study
CRISPRi-2	pCRISPathBrick, CRISPR spacer targeting *cysH* on open reading frame	This study


Plasmids used for CRISPRi/dCas9-mediated transcriptional repression was obtained from Addgene (Plasmid #65006) (Supplementary Figure S1) and constructed as previously reported (Cress et al., 2015). The specific targeting spacer of *cysH* from the *E. coli* K12 genomic DNA was identified near promoter (*cysH1*) and open reading frame region (*cysH2*) to prevent RNAP binding and elongation, respectively. The primer pairs crRNA1-Fw/Rv and crRNA2-Fw/Rv were used to construct CRISPRi-1 and CRISPRi-2 listed in Supplementary Table S1. Both primers were synthesized, phosphorylated with T_4_ polynucleotide kinase, and annealed (Cress et al., 2017). The products insert were then ligated into *Bsa*I-digested, dephosphorylated, gel-purified CRISPRi plasmid backbone. *E. coli* DH5α was used for cloning experiments. All CRISPRi plasmid arrays possessing synthetic the specific targeting spacer were verified by colony PCR with primer pairs cPCR-Fw/Rv (Supplementary Table S2) and sequencing. Plasmids were constructed with CRISPRi-1 and CRISPRi-2 were transformed into the wild-type strain through calcium chloride and a heat-shock method (Dagert and Ehrlich, 1979), forming variant S1 and S2 strains (**Table 1**).

### Culture Conditions and Recombinant Protein Expression

Wild-type *E. coli* was cultured in Luria Bertani (LB) liquid medium. The sample was kept in a 250 mL flask on a 37°C with shaking incubator 200 rpm. Kanamycin (50 μg mL^-1^) was used for plasmid selection and maintenance. A total of 250 μL of the pre-inoculum of wild-type *E. coli* was transferred to 50 mL fresh LB liquid medium containing an appropriate amount of antibiotic and then incubated at 37°C with 200 rpm shaking. Protein expression was induced by the different concentration of IPTG (0.1, 0.5, and 1.0 mM) when the optical cell density at 600 nm (OD_600__nm_) reached 0.6–0.8. The cell growth was continued at 20°C for 20 h and harvested by centrifugation at 842 × *g* for 15 min. The sample was washed twice with 50 mM Tris–HCl (pH 7.5) buffer containing 10% glycerol and prepared for sonication in 1 mL of the same buffer. Following centrifugation at 13,475 × *g* for 30 min at 4°C, the resulting soluble and insoluble protein fractions were analyzed by 12% sodium dodecyl sulfate-polyacrylamide gel electrophoresis (SDS-PAGE).

### Analysis of Cell Growth and mRNA-Expression Levels

The seed culture of the wild-type and S1, S2 strains were transferred to 50 mL fresh LB medium in a 250 mL flask and OD_600__nm_ of each flask was diluted to 0.1. All the culture containing chloramphenicol (34 μg mL^-1^) or kanamycin (50 μg mL^-1^) were growth under sharking at 37°C with 200 rpm shaking. After 6 h, 0.1 mM IPTG was added to induce the CRISPRi system and the growth was continued at 20°C with 200 rpm shaking for 48 h.

Total RNA was isolated by using RNeasy Plus Mini Kit (Qiagen, United States). The QuantiTect Reverse Transcription Kit (Qiagen, United States) was used to synthesize the cDNA from 1 μg total RNA sample. Power SYBR Green Master Mix (Thermo Fisher Scientific, United States) was performed by quantitative real-time PCR (qRT-PCR) on StepOnePlus real-time PCR system (Applied Biosystems, United States). The 16S rRNA gene was used to an endogenous control with primer pairs 16S rRNA-Fw/16S rRNA-Rv (Supplementary Table S2). The comparative 2^-Ct^ experiment was used to calculate relative gene expression (Livak and Schmittgen, 2001) by using wild-type as the reference sample with primer pairs *cysH*-Fw/*cysH*-Rv. The results were analyzed using StepOne Software v2.3 (Applied Biosystems, United States).

### Intracellular PAPS Collection and Quantification

Three recombinant strains including wild-type, S1 and S2 were grown, induced with 0.1 mM IPTG and expressed in 50 mL LB medium at 20°C with 200 rpm shaking for 12 h. Then, the cells were harvested by centrifugation, suspended, and incubated in 250 mL flasks containing 50 mL of M9 minimal salt medium pH 7.4 at 28°C for 12 h. After that, the samples were taken, chilled immediately on ice and centrifuged at 842 × *g* at 4°C for 15 min. One milliliter of 6% perchloric acid was used for cell lysis and 0.3 mL of 3 M potassium carbonate was added to neutralize while vortexing the sample (Fowler et al., 2009). After centrifugation removed the cell residue, the supernatant containing was collected, filtered through a 0.22-μm syringe filter and injected into UFLC-PDA to determine the products yield. The determination of microbial biomass was carried out by dry cell weight using a 0.22-μm syringe filter. An aliquot of 1 mL cell culture was filtered, washed with distilled water, and dried in a conventional oven. DCW was represented by the weight difference between empty membranes and those with cell residues (Wu et al., 2015).

### Sulfated Naringenin Production and Extraction

The wild-type and S1, S2 strains harboring heterologous a ST and CRISPRi were first expressed in 50 mL fresh LB medium by 0.1 mM IPTG at 20°C for 12 h. Next, the cells were harvested by centrifugation, washed twice with 100 mM phosphate buffer pH 7.4, and incubated at 28°C with shaking in 250 mL flasks containing 50 mL M9 minimal salt medium pH 7.4 (Yan et al., 2005). Appropriate antibiotics and IPTG were added at the same time. Subsequently, 100 μM substrate was supplemented to the same samples and kept on 48 h. We carried out fermentation in 3-L of optimal media under an optimal condition for large-scale production of naringenin derivatives. The processing of fermentation was followed as previously described (Chu et al., 2016). Furthermore, the culture samples were extracted with twice volume of ethyl acetate. The extracts were concentrated by freezing rotary evaporator, suspended in methanol, and then injected into UFLC-PDA to determine the products yield.

### HPLC Analysis

The UFLC-PDA system containing a reversed-phase column [Mightysil RP-18 GP 250-4.6 (5 μm) Cica-Reagent; Kanto Chemical Co., Inc., Japan] was used to separate the samples. The mobile phase containing solvent A (HPLC-grade water consisting 0.05% TFA) and solvent B (100% HPLC-grade methanol) was maintained at 30°C. A calibration PAPS and naringenin standards were created with various concentration.

For quantification of PAPS, the intracellular PAPS was analyzed at a UV absorbance of 254 nm (Uesugi et al., 1997; Xu et al., 2012). The mobile phase including solvents A and B was maintained at 1 mL min^-1^ for 10 min. The program was followed by 10% B for 1 min, 20% B for 1 min, 30% B for 30 s, 35% B for 30 s, 40% B for 1 min, 50% B for 4 min, 90% B for 1 min, and 10% B for 1 min.

Quantification of production was based on the peak areas obtained at 290 nm. The conversion percentage of the substrate was determined after integrating substrate and product peaks. Solvents A and B were run at 1 mL min^-1^ for a 30 min. The gradient of the mobile phase was carried out and followed by 20% B for 5 min, 50% B for 5 min, 70% B for 5 min, 90% B for 5 min, and 10% B for 10 min.

The purification of naringenin derivatives was performed by preparative HPLC (Shimadzu, Tokyo, Japan) with C_18_ column [YMC-Pack ODS-AQ (150 mm × 20 mm I.D., 10 μm)] connected to a UV detector at 290 nm using a 40 min binary program with 5% B for 5 min, 40% B for 5 min, 40% B for 5 min, 90% B for 5 min, and 10% B for 10 min at a flow rate of 10 mL min^-1^.

### Mass Spectrometry and Nuclear Magnetic Resonance

High-resolution quadruple time-of-flight electrospray ionization-mass spectrometry (HR–QTOF ESI/MS) analysis was carried out with an ACQUITY column coupled an SYNAPT G2-S (UPLC, Waters Corp., Billerica, MA, United States). A reversed-phase column [Acquity BEH C_18_ 2.1 mm × 100 mm (1.7 μm)] was used to separate the samples. ESI/MS detection of the samples: positive ion mode ESI^+^, acquisition range: 50–1,400 *m/z*, capillary voltage: 2.5 kV, cone voltage: 30 V, source temperature: 120°C, desolvation gas temp: 600°C, cone gas flow: 20 L h^-1^, desolvation gas flow: 800 L h^-1^. The analyses were performed at a flow rate of 0.35 mL min^-1^ using the same mobile phase with a gradient of 30% B for initial, 90% B for 4 min, 100% B for 3 min, 100% B for 2.5 min, and 37.5% B for 2.5 min. MassLynx software version 4.1 (Waters Corp.) was used for analysis the chromatograms.

The purified products were dried, lyophilized, and recorded on Bruker Biospin 300 MHz spectrometer in DMSO-*d_6_* for proton ^1^H-NMR.

### Antibacterial Activity

Nine Gram-positive bacteria and six Gram-negative bacteria listed in **Table 2** were used for testing the antibacterial of naringenin and its derivative. All strains were cultured in LB medium at 37 ± 1°C. We applied the paper disc diffusion method using ampicillin (current antibiotic standard) as an antibacterial positive control for screening the antibacterial agent (Rios et al., 1998). Each inoculates consisted of 10^6^ colony forming units (CFU mL^-1^) and was spread on MHA plates for bio-assay. Sterile filter paper discs (6 mm in diameter) consisting of 10 μL of 100 mM of compounds dissolved in MeOH were spotted on the agar surface. The plates were incubated at 37 ± 1°C and checked for 36 h.

**Table 2:** Inhibition zone diameter (mm) of naringenin and naringenin 7-sulfate against nine Gram-positive bacteria and six Gram-negative bacteria.

No.	Pathogens	Naringenin	Naringenin 7-sulfate	Ampicillin
**Gram-positive bacteria**				
1	*S. aureus* CCARM 0205 (MSSA)	–	7.0 ± 0.08	7.2 ± 0.09
2	*S. aureus* CCARM 0204 (MSSA)	–	+	+
3	*S. aureus* CCARM 3634 (MRSA)	–	+	23 ± 0.27
4	*Proteus hauseri* NBRC 3851	–	+	20 ± 0.24
5	*Micrococcus luteus* KACC 13377	–	9.5 ± 0.18	15 ± 0.36
6	*Bacillus subtilis* ATCC 6633	–	–	–
7	*Bacillus subtilis* KACC 17047	–	–	–
8	*Enterococcus faecalis* 19433	–	–	13 ± 0.18
9	*Enterococcus faecalis 19434*	–	–	14 ± 0.20
**Gram-negative bacteria**				
10	*Kocuria rhizophila* NBRC 12708	–	–	16 ± 0.14
11	*Klebsiella pneumoniae* ATCC 10031	–	–	16 ± 0.12
12	*E. coli* ATCC 25922	–	–	30 ± 0.32
13	*Salmonella enterica* ATCC 14028	+	+	13 ± 0.14
14	*Pseudomonas aeruginosa* KACC 10232	–	7.2 ± 0.12	–
15	*Enterobacter cloacae* subsp. *dissolvens* KACC 13002	–	+	9 ± 0.10


*-, no inhibition zone; +, inhibition zone detected.*

### Anticancer Activities

Three cancer cell lines containing A375SM melanoma, MCF-7 breast cancer, AGS gastric cancer, and 267B1 cellosaurus were observed from the Korean Cell Line Bank (KCLB, Seoul, South Korea). Minimum essential medium (MEM) added 10% fetal bovine serum (FBS) (Gibco, Grand Island, NY, United States) was used for culture the cell lines at 37°C in a humidified 5% CO_2_ incubator. Cells seeded at 2 × 10^3^ cell well^-1^ in 96-well plates (SPL Life Sciences, Gyeonggi, South Korea) were treated with each compound in serial dilution (400, 200, 100, and 50 μM) for 72 h. Taxol, a current anticancer agent, was also carried out to provide an additional point of comparison. We examined cell growth using an MTT colorimetric assay (Lee et al., 2017).

### Statistical Analysis

Values are mean ± standard deviation (SD). All the results were the average of three independent experiments with SD (*n* = 3). Differences with *p*-values <0.05 and <0.005 were indicated a considered significant.

## Results

### *Escherichia coli* Expression of Recombinant *A. thaliana* SULTs

To optimize conditions for the expression of recombinant ST At2g03770 from *A. thaliana*, we induced with various IPTG concentrations (0.1, 0.5, and 1.0 mM) under 20°C in LB liquid medium. The SDS-PAGE analysis of the soluble and insoluble fraction of At2g03770 indicated the highest expression level as determined at 0.1 mM IPTG. The recombinant protein, At2g03770 (37.72 kDa), was overexpressed in *E. coli* BL21 (DE3) and obtained in the soluble fraction (Supplementary Figure S2).

### Construction of CRISPRi System in *E. coli*

**FIGURE 2:**
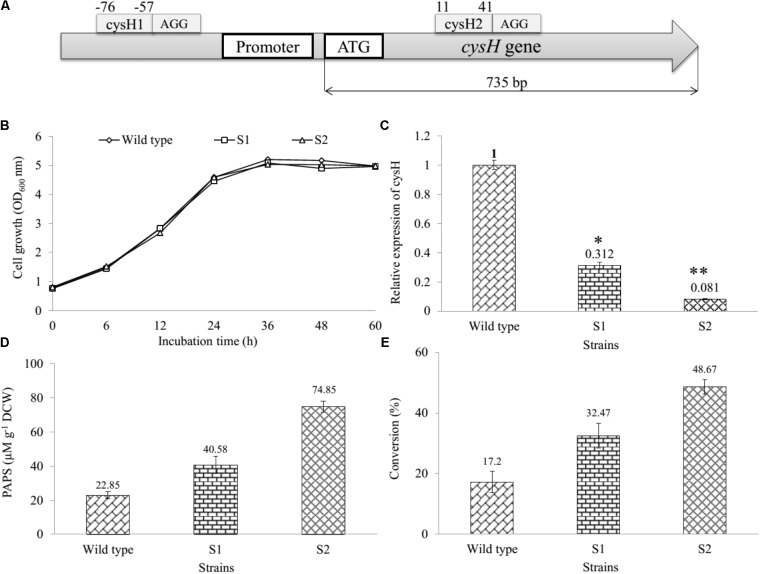
Establishment and assessment of CRISPRi in sulfur metabolic pathway of *E. coli*. **(A)** The binding at different position on *cysH* gene using CRISPRi system. Sites selected *cysH1* (from position –76 to –57) after the PAM sequence AGG and *cysH2* (from position 11 to 44) after PAM sequence AGG. **(B)** Comparison of cell growth in LB broth medium at OD_600_, **(C)** qRT-PCR analysis, **(D)** concentration of PAPS, and **(E)** the percentages of bioconversion between recombinant engineered *E. coli* carried out CRISPRi system targeting *cysH* gene and wild-type (^∗^*p* < 0.05, ^∗∗^*p* < 0.005).

Gene *cysH* was selected for studying CRISPRi system on the sulfur metabolism of *E. coli.* The knockdown of *cysH* might to the increase of the intracellular PAPS pool enhancing production of sulfated substrates. Therefore, two 66 bp complementary offset oligonucleotides containing 30 bp protospacer (target) sequence were designed (Supplementary Table S1) and inserted to pCRISPathBrick, resulted in CRISPRi-1 and CRISPRi-2 systems formation (**Table 1**). The length of the *cysH* gene is 735 bp, while the length of the target sequence is 30 bp. To transcriptional interference, *cysH1* was designed 56 bp upstream from start codon (from position -76 to -57), after the PAM sequence AGG (from position -58 to -56). At the same time, *cysH2* was designed 10 bp downstream of the start codon (from position 40 to 11), after PAM sequence AGG (from position 41 to 43) (**Figure 2A**). The unique properties of *E. coli* BL21 genomic of each two protospacer were confirmed *via* nucleotide BLAST^1^. The plasmids were assembled and then biotransformation into *E. coli* DH5α. Colony PCR (cPCR) was performed with cPCR-Rv and cPCR-Fw primers (Supplementary Table S2). The cPCR products were confirmed through 2% agarose gel with 50 bp DNA ladder marker (ELPIS-Biotech. Inc., South Korea). The length of amplifying obtained from clones of pCRISPathBrick is 85 bp, while a 66 bp increase in PCR obtained from clones of CRISPRi-1 and CRISPRi-2 (Supplementary Figure S3). The plasmids CRISPRi-1 and CRISPRi-2 were transformed into wild-type strain, forming production strains S1 and S2, respectively (**Table 1**).

### Effect of CRISPRi System on Cell Density and Gene Expression

We determined and compared the growth curves of wild-type, S1, and S2 strains by using a spectrophotometer (Thermo Fisher Scientific, United States) *via* the OD_600 nm_. The data showed that the S1 and S2 had to resemble with the wild-type on the rate of cell growth (**Figure 2B**), indicating that *cysH* gene inhibition by CRISPRi did not affect the cell growth. In addition, the relative qRT-PCR data of knockdown S1 and S2 strains were analyzed through *cysH* mRNA quantification using 16S rRNA as an endogenous control and wild-type as the reference sample. 16S rRNA, known as a housekeeping gene, is one of the most commonly used endogenous control in *E. coli* (Zhou et al., 2011). The data showed qRT-PCR cycle threshold values for 16S rRNA gene expressed at almost similar levels in all three samples containing wild-type, S1, and S2 with 23.852 ± 0.371, 22.558 ± 0.031, and 21.732 ± 0.115, respectively. This results demonstrated that the suitability of 16S rRNA gene for the specific case of this experiment (Supplementary Figure S4). Importantly, the value of relative expression of *cysH* gene transcription in wild-type was 1, while this figure for S1 and S2 were 0.312 and 0.081, respectively (**Figure 2C**). The result indicated that both the prevent RNA polymerase binding (CRISPRi-1) and elongation (CRISPRi-2) have high efficiency of the reducing transcriptional expression level of *cysH*.

### The Knockdown of *cysH* Gene Increased PAPS and Naringenin Derivatives

The intracellular PAPS in three strains were extracted using the method described above. All the samples consisting authentic ADP, ATP, and PAPS standard were determined by using UHPLC-PDA coupled HR-QTOF ESI/MS. UHPLC-PDA analysis showed the ADP standard appeared at retention time *t*_R_ ∼ 0.83 min, while *t*_R_ of ATP and PAPS standard were 2.71 and 1.27 min. Interestingly, the intracellular PAPS was detected at *t*_R_ ∼ 1.27 min in the cytosol of wild-type as well as mutant strains (Supplementary Figure S5). These peaks were confirmed by HR-QTOF ESI/MS and the results were shown in Supplementary Table S3 and Supplementary Figures S6, S7. These results suggested that UHPLC-PDA coupled HR-QTOF ESI/MS not only the distinguishing between PAPS from potential interferents ADP and ATP but also the detection of the intracellular PAPS in the cytosol of *E. coli*. Finally, the intracellular concentration of PAPS was analyzed and shown in **Figure 2D**. While the figure for S1 and S2 strains went up to 40.58 and 74.85 μM g^-1^ DCW, the concentration of PAPS for wild-type obtained a 22.85 μM g^-1^ DCW at the same time. This result demonstrated that *cysH* is the most required target to inhibit the PAPS consumption.

Moreover, we used the three recombinant strains to produce the sulfated derivative from naringenin. The UFLC-PDA chromatograms of extract from the whole cell of all strains showed a new peak at retention time *t*_R_ ∼ 18.248 min (P1) in comparison with naringenin standard at *t*_R_ ∼ 19.979 min under UV absorbance at 290 nm (Supplementary Figure S8). These peaks were further analyzed by HR-QTOF ESI/MS. The found mass of naringenin was ∼273.0780 [M + H]^+^ *m/z*^+^ equivalent to molecular formula C_15_H_13_O_5_ with λ_max_ ∼ 287 nm, for which calculated mass was ∼273.0763 (Supplementary Figure S9A). The found mass of P1 at ∼353.0330 with λ_max_: 277; 335 nm, corresponding to the exact mass of the mono-sulfate derivative of naringenin with molecular formula C_15_H_12_O_8_S for [M + H]^+^ *m/z*^+^ ∼ 353.0331 (Supplementary Figure S9B). The structural identifies of the product could be verified *via* NMR in the future experiment. The percentages of bioconversion of naringenin to mono-sulfated naringenin were 17.2% in the wild-type strain, while the figure for both S1 and S2 strains showed an enhancement to 32.47 and 48.67% at the same time (**Figure 2E**). These results demonstrated that S1 and S2, both mutant strains include the knockdown of the *cysH* gene were used for the improvement of naringenin derivative. It might be true that S2 could be a good recombinant host system produce derivatives product form naringenin.

### Regulating the Concentration of Inorganic Sulfate and Glucose in Medium

**FIGURE 3:**
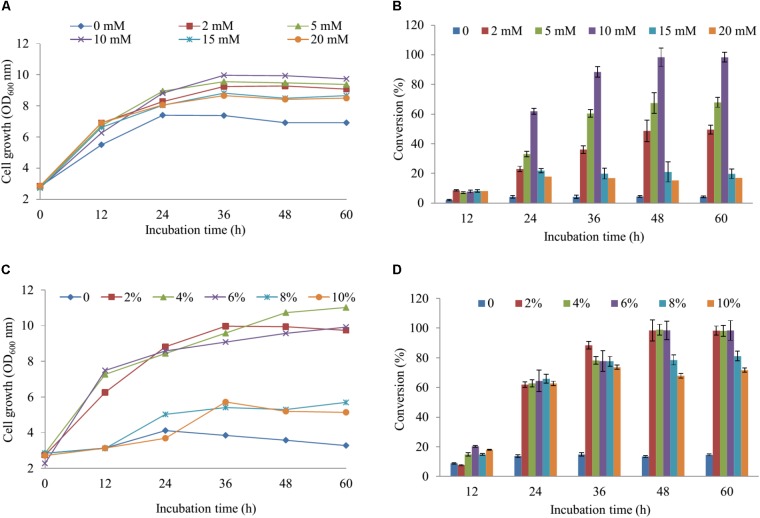
The regulation of the concentration MgSO_4_ and glucose in M9 media. The concentration of $SO_{4}^{2-}$ effect on cell growth **(A)** and the production sulfated naringenin **(B)**. The percentages of glucose influent into cell growth **(C)** and bioconversion of naringenin to its derivative at the various incubation time **(D)**.

For regulation of the concentration MgSO_4_, we used the M9 minimum media including the various concentrations of MgSO_4_ (2, 5, 10, 15, and 20 mM) in comparison with the M9 medium without MgSO_4_. The cell growth and substrate conversion were taken out at 12 h intervals. While the production was noticeably low in the medium without MgSO_4_, the maximum bioconversion of substrate obtained 98.34% at 48 h with OD_600_ ∼ 9.94 in M9 medium with 10 mM MgSO_4_ when 100 μM naringenin was added (**Figures 3A,B**). Subsequently, the production was carried out by using an M9 medium with 10 mM MgSO_4_ consisting of the various concentrations of glucose (2, 4, 6, 8, and 10%). The data showed that nearly 100% of 100 μM naringenin was converted to its sulfated derivatives during the addition 2, 4, and 6% glucose at 48 h with OD_600_ ∼ 9.94, 10.73, and 9.57, respectively (**Figures 3C,D**). The M9 medium supplementation of 10 mM MgSO_4_ and 2% glucose were selected for further optimizing the concentration of substrate.

### Bioconversion With Different Naringenin Concentration and Scale-Up by Fermentation

**FIGURE 4:**
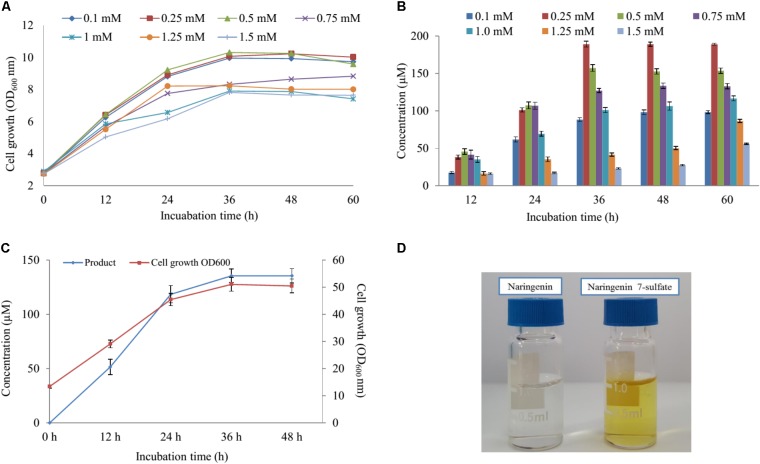
The effect of substrate concentration and scale-up production. **(A)** Cell growth at OD_600__nm_ and **(B)** the substrate concentration optimization of naringenin in biotransformation. **(C)** Cell growth at OD_600__nm_ and the scale-up production sulfated naringenin in 3-L fermentation at different time intervals. **(D)** Comparison of color between substrate and product.

Five various concentration of naringenin (200, 400, 600, 800, and 1,000 μM) were supplied for biocatalytic reaction system with the S2 strain. The OD_600_ and conversion rate of naringenin to its derivative were monitored at 12-h intervals. The highest production obtained 189 μM at 48 h with OD_600_ ∼ 10.24 when 250 μM was fed under M9 medium including 10 mM MgSO_4_ and 2% glucose (**Figures 4A,B**).

Finally, these optimal conditions were applied for the bioconversion process into the 3-L fermenter (Biotron, South Korea). When OD_600_ ≥ 6, 0.1 mM IPTG was induced and the temperature decreased to 20°C. After 12 h induction, 250 μM (∼264 mg 3-L) of naringenin was fed to the culture under the pH and temperature were maintained at 7.4 and 30°C, respectively. The samples were taken at 12 h interval and measured by UFLC-PDA. UFLC-PDA analysis revealed that sulfated naringenin was obtained to 135.49 μM (∼143.1 mg 3-L) at 36 h with OD_600_ ∼ 51.06 (**Figure 4C**).

### Structural Elucidation of Sulfated Naringenin

Previous study exhibited that even though no sulfating activity toward 5-, 3′ and 4′-hydroxyflavone, At2g03770 showed the catalytic activity of 7-hydroxyflavone (Hashiguchi et al., 2013). We re-confirmed the structural compound by ^1^H-NMR of naringenin standard and purified sulfated product at 300 MHz in DMSO-*d_6_*. The ^1^H-NMR spectrum of sulfated naringenin displayed the absence of OH- group signal at δ = 10.83 ppm for C-7 in comparison with the ^1^H-NMR spectrum of naringenin (Supplementary Figure S10). Moreover, the ^1^H-NMR spectrum of this compound exhibited a lower shift at H-6 and H-8 (Supplementary Table S4), indicating that the hydroxyl group at 7 positions of naringenin was substituted by a sulfate group. These data also agreed well with naringenin 7-sulfate obtained in the fungus *Cunninghamella elegans* NRRL 1392 (Ibrahim, 2000). Based ppm all the results, we could be identified that product was naringenin 7-sulfate. While naringenin was colorless compound, naringenin 7-sulfate was obtained as a yellowish solid (**Figure 4D**).

### Antibacterial and Anticancer of Compounds

Results of disc diffusion assays displayed that naringenin showed only antibacterial activity against *Salmonella enterica* ATCC 14028 when 10 μL of 100 mM compound was used. In contrast, naringenin 7-sulfate exhibited broad-spectrum antibacterial activity against not only Gram-positive bacteria containing *Staphylococcus aureus* CCARM 0205, *S. aureus* CCARM 0204, *S. aureus* CCARM 3634, *Proteus hauseri* NBRC 3851, *Micrococcus luteus* KACC 13377, but also negative–positive bacteria consisting of *S. enterica* ATCC 14028, *Pseudomonas aeruginosa* KACC 10232, and *Enterobacter cloacae* subsp. *dissolvens* KACC 13002. Noticeably, naringenin 7-sulfate exhibited antibacterial activity against *M. luteus* KACC 13377 with a zone of inhibition values of 9.5 ± 0.18 mm. Moreover, we detected a zone of inhibition against *S. aureus* CCARM 0205 (MSSA) by naringenin 7-sulfate and ampicillin seems to share the most similarity with values of 7.0 ± 0.08 and 7.2 ± 0.09 mm. Interestingly, while naringenin and ampicillin did not show antibacterial activity against *P. aeruginosa* KACC 10232, naringenin 7-sulfate displayed a zone of inhibition values of 7.2 ± 0.12 mm (**Table 2** and Supplementary Figure S11). These results indicated that sulfation of naringenin at hydroxyl group of C-7 position could be advantageous for intensifying its antibacterial activity against various bacteria.

**FIGURE 5:**
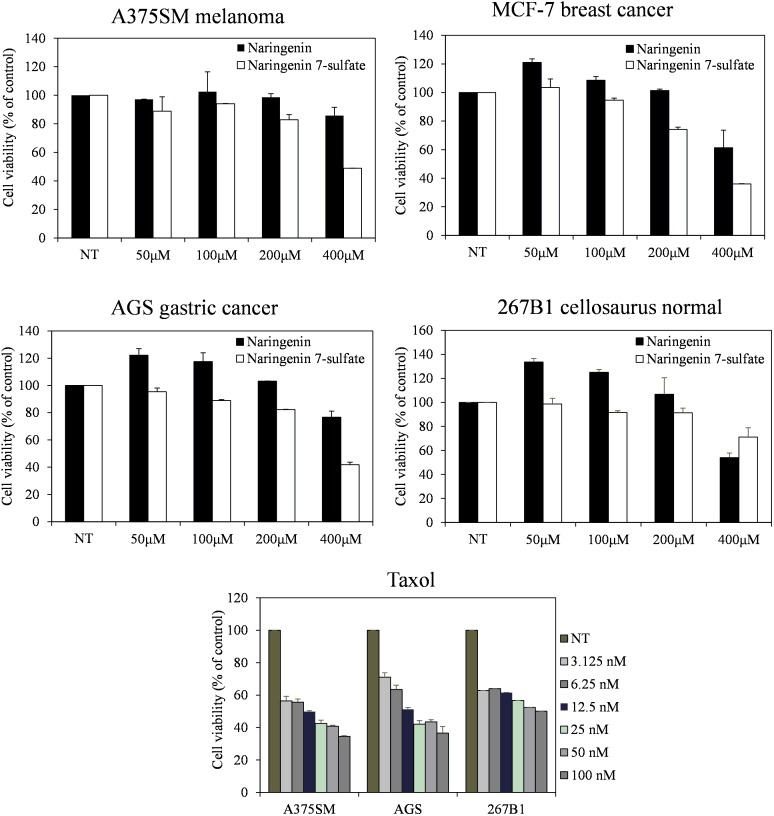
Effect of naringenin and naringenin 7-sulfate on the cell viability of three cancer cell lines.

Furthermore, the cell viability data showed that naringenin 7-sulfate exhibited good anticancer activities where naringenin did not have anticancer activities against all cell lines. Cell viability of A375SM, MCF-7, AGS, and 267B1 treated 400 μM of naringenin 7-sulfate reduced approximately 48.85, 35.96, 41.78, and 71.20% (*p* < 0.05), respectively, in comparison with normal test (NT) (**Figure 5**). These results suggest that naringenin 7-sulfate was relatively less cytotoxic to non-cancer cell than cancer cell lines. In addition, the data showed naringenin 7-sulfate was less cytotoxic than naringenin in the non-cancer cell at 400 μM. As expected, taxol exhibited highly strong anticancer activity at nanomolar ranges of concentration. Taxol also inhibited A375SM and AGS cancer cell lines a little better than 267B1 non-cancer cell line with inhibition of 65.47, 63.40, and 49.90% at 100 nM, respectively (**Figure 5**). In conclusion, naringenin 7-sulfate could be the more potent anticancer agent with lower cytotoxicity against non-cancer cell than naringenin.

## Discussion

Sulfotransferases can be found in various life form from prokaryotes to eukaryotes (Suiko et al., 2017). In a model plant organism, *A. thaliana*, 18 different STs classified into seven groups are present (Klein and Papenbrock, 2004). Among all the *A. thaliana* STs, At2g03770 has been exploited as catalysis the sulfation for a variety of flavonoids (Hashiguchi et al., 2013). In this research, we produced 17.2 μM naringenin derivatives *via* overexpressed At2g03770 in *E. coli*. Although the yield of substantial amount of At2g03770 product appears in insoluble fraction might be led to the lower harvest of sulfated product (Supplementary Figure S2), the result indicated that heterologous expression STs from *A. thaliana* could be efficient produce sulfated compounds in whole cell biotransformations. Moreover, metabolic engineering in *E. coli* also might be applied for the enhanced production of prospective compounds extensively.

In *E. coli* sulfur metabolism, operon *cysUWAsbp* codes for a sulfate permease, an ATP binding cassette (ABC)-type transporter (Sekowska et al., 2000), carried the sulfate into the internal cell. Subsequently, ATP sulfurylase coded by operon *cysDN*, catalyzed an adenylylation of sulfate in the form of APS. After being activated by APS kinase *cysC*, APS is phosphorylated to PAPS (Hatzios and Bertozzi, 2011). PAPS is the universal sulfate donor in the reaction of sulfation performed by STs on the bacterial metabolite. On another hand, consumption of PAPS in sulfate assimilation pathway start catalyzed with the *cysH*-encoded PAPS ST. This reaction is continuously reduced for the biosynthesis of essential reduced sulfur metabolites (**Figure 1**). Therefore, CRISPRi has been introduced into background strain consist of expressed ST At2g03770, targeting strategy to inhibit PAPS consumption and subsequently increase sulfated naringenin. This is the first time the production of sulfated flavonoids has published with the CRISPRi system in *E. coli*.

CRISPRi recently has applied to the enhanced biosynthesis of flavonoid (Cress et al., 2015) and *O*-methylated anthocyanin (Cress et al., 2017) through transcription regulation of metabolic pathway in *E. coli*. Compared to the traditional gene deletion methods, CRISPRi showed the great tool for targeted gene inhibition in microorganisms. Firstly, the construction of CRISPRi plasmid is simple and saving time because dCas9 protein and sgRNA expressed in one vector (Supplementary Figure S1). The efficiency of two different nucleotide sequence targeted on the *cysH* gene was not the same. The proportion suppression of spacer sequence bound to open reading frame region (*cysH2*) was 1.34-fold higher than the figure for spacer sequence bound to near promoter (*cysH1*), at 91.9 and 68.8%, respectively (**Figure 2C**). The reason behind the variation in gene expression level could be the corresponding to the distance of *cysH2* and *cysH1* to transcription start site, 10 bp of *cysH2* in compared with 56 bp of *cysH1* (**Figure 2A**). Moreover, the regulation of sulfate metabolic pathway by CRISPRi system result in improving PAPS pool without affecting cell growth (**Figure 2B**). Ultimately, CRISPRi system not only reduces the metabolic burden associated with high-copy overexpression of heterologous ST At2g03770, but also increase production of target molecules. In the whole cell biocatalysis system, production of naringenin derivatives increased by up to 2.83-fold (**Figure 2E**). This result suggested that S2 strain not only led to the accumulation of intracellular PAPS but also improved the efficiency of sulfation of naringenin.

Recently, the analysis of polar molecules such as ATP, ADP, and PAPS was carried out *via* liquid chromatography-mass spectrometry (Johnsen et al., 2011; Dowood et al., 2016). In this study, UHPLC-PDA coupled HR-QTOF ESI/MS allowed the detection PAPS, although the quantity of PAPS was lowed in the cytoplasm of *E. coli.* The method not only is high accuracy but also simple to perform with general solvent and saving time. Moreover, this method was very significant for the separation between PAPS and nucleoside triphosphates as ATP and ADP, because these compounds were the same physical properties (Dowood et al., 2016).

The cultivation of the wild-type and S1, S2 strains was the initial phase of rapid cell growth at 37°C, followed by a growth arrest phase at 20°C, and the biosynthesized compound produced at 28°C. The growth-arrested *E. coli* induced by using low temperature led to improved the conformational quality and the solubility of STs from *A. thaliana* and CRISPRi systems (Vera et al., 2007; Cress et al., 2015), which associated with an increased sustained viability and an extended production phase (Rosano and Ceccarelli, 2014). This experiment has a significant impact on the optimal production of the target compound. We decided to the production sulfated naringenin in M9 medium containing MgSO_4_ and glucose as sulfur and carbon sources, respectively. Almost 100% of conversion rate from a substrate to its derivative was obtained in media consisting of 10 mM MgSO_4_ and 2–6% glucose, indicating that sulfated compound all accumulated in the extracellular fraction. One possible reason behind this result could be organic anion molecules as sulfated naringenin has been eliminated into extracellular by *E. coli* multidrug resistance ATP binding cassette transporters (Chang and Roth, 2001). On the other hand, sulfated naringenin including the negative charged $SO_{4}^{2-}$ led to might not to cross the cell membrane of *E. coli*. Furthermore, even though the media lacking MgSO_4_ resulted in the cell growth was low, the production still obtained around 4.42% (**Figures 3A,B**). The reason could be because of when the growth medium absent inorganic sulfur, *E. coli* can induce a series of sulfate starvation-inducible genes led to utilize organosulfur compound as a source of sulfur (van der Ploeg et al., 2001). Moreover, the media absence glucose also caused the production was low as well as the cell growth inefficient (**Figures 3C, 4D**). These results demonstrated that both $SO_{4}^{2-}$ and glucose are an essential factor produce sulfated naringenin by At2g03770-expressed in *E. coli* cells. However, the high concentration of $SO_{4}^{2-}$ (above 15 mM) and glucose (over 6%), as well as more than 0.5 mM of the substrate, could be a reason for inhibition of sulfation product in *E. coli* whole cell (**Figures 3, 4**). Finally, the engineered *E. coli* S2 strain has been applied successfully for the large-scale production of naringenin 7-sulfate, which obtained at 135.49 μM [∼143.1 mg (47.7 mg L^-1^)] in a 3-L fermenter (**Figure 4C**). These results indicate that the system is efficient and could be applied for other flavonoids to generate the libraries of molecules with various sulfation approaches.

We also evaluated the antibacterial activity of naringenin and its derivative. Five Gram-positive bacteria *S. aureus* CCARM 0205, *S. aureus* CCARM 0204, *S. aureus* CCARM 3634, *P. hauseri* NBRC 3851, *M. luteus* KACC 13377 and three Gram-negative bacteria *S. enterica, P. aeruginosa, E. cloacae* were sensitive with naringenin 7-sulfate. This result indicated that negatively charged sulfate group might be improved to naringenin for antibacterial activity, however, the mechanism of this compound against bacterial pathogens have been not reported. In addition, naringenin derivative exhibited the most potential anticancer activity against three tested cancer cell lines. The compound showed the similarity in features between its and taxol the less cytotoxic to non-cancer cell line in comparison with cancer cell lines (**Figure 5**). This is the first report of the activity of naringenin 7-sulfate against A375SM, MCF-7 and AGS cancer cell lines. The previous research has shown that the substituent at the C-7 position of naringenin containing thiophenecarboxylate, phenyl carbonate, isobutyrate, methyl benzoate, and allyloxy inhibited effects on HCT116 human colon cancer cell line *via* block G1 cell cycle progression by interaction with cyclin-dependent kinase 2 (CDK2) (Yoon et al., 2013). This possible mechanism behind the anticancer activity of naringenin 7-sulfate against three tested cancer cell lines, however, the exact mechanisms of action of this compound must confirm in further studies.

In summary, we targeted to improving production of sulfated flavonoids in engineered *E. coli*. This is the first report on CRISPRi mediated inhibition in the sulfur metabolism of *E. coli*. Repression of key reduced sulfur metabolite enzyme *cysH* by over 91%, causing increase intracellular PAPS accumulation and enhancement of naringenin 7-sulfate over 3.28- and 2.83-fold compared to control, respectively. Further media culture optimization led to obtained at 135.49 μM [∼143.1 mg (47.7 mg L^-1^)] in a 3-L fermenter. This engineered *E. coli* opened prospects for the biosynthesis of the sulfated flavonoids. In addition, naringenin 7-sulfate exhibited antibacterial activity against both Gram-positive and Gram-negative bacteria. This compound could be also used as a cancer drug when shown to anticancer activity against A375SM melanoma, MCF-7 breast, and AGS gastric cancer cell lines.

## Author Contributions

LC designed, performed the majority of the experiment work, analyzed data, and wrote the manuscript. DD and TY helped in analyzing data. HS and HJ did the majority of anticancer activities. JS and LC were responsible for the original concept and supervised the work. All authors read and approved the final manuscript.

## Conflict of Interest Statement

The authors declare that the research was conducted in the absence of any commercial or financial relationships that could be construed as a potential conflict of interest.

## Footnotes

1 https://blast.ncbi.nlm.nih.gov
