# Supplementary material for: Metabolic Engineering of Escherichia coli for Enhanced Production of Naringenin 7-Sulfate and Its Biological Activities
Source: Front Microbiol. 2018 Jul 27;9:1671. doi: 10.3389/fmicb.2018.01671 (PMC6072979; doi:10.3389/fmicb.2018.01671)
Supplement: Supplementary file 1 [file Data_Sheet_1.PDF]

Supplementary Material

Metabolic Engineering of *Escherichia coli* for Enhanced Production of Naringenin 7-sulfate and Its Biological Activities

Luan Luong Chu

chuluongluan218@gmail.com

Dipesh Dhakal

medipesh@gmail.com

Hee Jeong Shin

gmlwjd903@naver.com

Hye Jin Jung

poka96@sunmoon.ac.kr

Tokutaro Yamaguchi

yamaguchi@sunmoon.ac.kr

*** Correspondence:**
Corresponding Author: Prof. Jae Kyung Sohng
sohng@sunmoon.ac.kr

Table S1. CRISPRi oligonucleotides used in this study

ID	Nucleotide Sequence (5'→3')	[Proto]spacer Sequence (5'→3')<br>Reverse complement of spacer sequence	PAM (5'→3')
crRNA1-Fw	AAACTTGTGGGATTAACCATCAGCCCGGTCTTGT GTTTTAGAGCTATGCTGTTTTGAATGGTCCCA	TTGTGGGATTAACCATCAGCCCGGTCTTGT	AGG
crRNA1-Rv	GTTTTGGGACCATTCAAAACAGCATAGCTCTAAA ACACAAGACCGGGCTGATGGTTAATCCCACAA	ACAAGACCGGGCTGATGGTTAATCCCACAA	
crRNA2-Fw	AAACTCGATCTAAACGCCCTGAACGAACTGCCG AGTTTTAGAGCTATGCTGTTTTGAATGGTCCCA	TCGATCTAAACGCCCTGAACGAACTGCCGA	AGG
crRNA2-Rv	GTTTTGGGACCATTCAAAACAGCATAGCTCTAAA ACTCGGCAGTTCGTTCAGGGCGTTTAGATCGA	TCGGCAGTTCGTTCAGGGCGTTTAGATCGA	

Table S2. Nucleotide sequences of primers

Primer	Nucleotides sequence (5'→3')
cPCR-Fw	GACAAAAATAGTCTACGAGGTTTTAG
cPCR-Rv	GAGTCCTATGAGCTTCCGAGACAG
16sRNA-Fw	AATGCGTAGAGATCTGGAGG
16sRNA-Rv	AAGGGCACAACCTCCAAGTC
cysH-Fw	CAACCTGAAAGTGTACCGTG
cysH-Rv	CATTCAGTTCTTTCAGAGCC

Table S3. UHPLC-PDA, the UV maxima absorbance, HR-QTOF ESI/MS analysis and molecular formula of ADP, ATP, PAPS standard and PAPS cytosol obtained from cell lysis of engineered *E.coli* strains.

**Product ions**	**UPLC-PDA(t_R)**	**UV maxima** (nm)	**Exact mass m/z$^+$~**	**Found mass m/z$^+$~**	**Molecular formula**
**ADP standard**					
$[M+H]^+$	1.07	256.7347	428.0367	428.0368	$C_{10}H_{16}N_5O_{10}P_2$
$[M+Na]^+$			450.0186	450.0178	$C_{10}H_{15}N_5NaO_{10}P_2$
**ATP standard**					
$[M+H]^+$	2.71	256.7347	508.0030	508.0030	$C_{10}H_{17}N_5O_{13}P_3$
$[M+Na]^+$			529.9850	529.9824	$C_{10}H_{16}N_5NaO_{13}P_3$
**PAPS standard**					
$[(M-C_5H_{12}O_{13}P_2S)+H]^+$	1.27	256.7347	136.0618	136.0618	$C_5H_6N_5$
$[M-SO_3OH)+H]^+$			410.0261	410.0266	$C_{10}H_{14}N_5O_9P_2$
$[(M-SO_3H)+H]^+$			428.0367	428.0374	$C_{10}H_{16}N_5O_{10}P_2$
**PAPS cytosol**					
$[(M-C_5H_{12}O_{13}P_2S)+H]^+$	1.27	256.7347	136.0618	136.0606	$C_5H_6N_5$
$[(M-H_2PO_3)+H]^+$			348.0704	348.0697	$C_{10}H_{15}N_5O_7P$
$[(M-SO_3H)+H]^+$			428.0367	428.0356	$C_{10}H_{16}N_5O_{10}P_2$
$[(M-2H+2Na)+H]^+$			552.9652	552.9225	$C_{10}H_{15}N_5Na_2O_{13}P_2S$

Table S4. Comparison of ^{1}H–NMR between naringenin standard and sulfated product

Carbon No	Naringenin	Sulfated product
2	5.43 (dd, *J* = 12.8, 2.9 Hz)	5.45 (dd, *J* = 12.8, 3.0 Hz)
3-*trans*	3.29 (m, *J* = 17.1, 12.8 Hz)	3.30 (m, *J* = 17.1, 12.8 Hz)
3-*cis*	2.67 (dd, *J* = 17.1, 3.0 Hz)	2.71 (dd, *J* = 17.1, 3.0 Hz)
5-OH	12.16 (dd, *J* = 1039.2, 81.7 Hz)	12.16 (s)
6	5.88 (s)	5.95 (s)
7-OH	10.83 (s)	
8	5.88 (s)	5.95 (s)
2′	7.31 (d, *J* = 8.6 Hz)	7.32 (d, *J* = 8.5 Hz)
3′	6.79 (d, *J* = 8.5 Hz)	6.79 (d, *J* = 8.5 Hz)
4′-OH	9.63 (s)	9.64 (s)
5′	6.79 (d, *J* = 8.5 Hz)	6.79 (d, *J* = 8.5 Hz)
6′	7.31 (d, *J* = 8.6 Hz)	7.32 (d, *J* = 8.5 Hz)
s–singlet, d–doublet, dd–doublet of doublet, m–multiplet.		

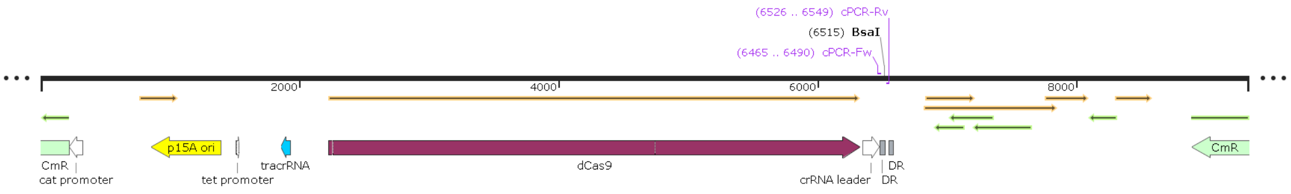

Figure S1. CRISPRi Plasmid used in this study. pCRISPathBrick vector (9236 bp) for expression of *S. pyogenes* dCas9, tracrRNA, crRNA leader, and two direct repeat possessing a single BsaI site for inserting user-defined spacer-repeat bricks. Cm^R, chloramphenicol resistance gene; p15A ori, the medium-copy numer p15A origin of replication; tet promoter, *E.coli* promoter for tetracycline efflux protein gene; cat promoter, promoter of *E.coli* cat gene encoding chloramphenicol acetyltransferase.

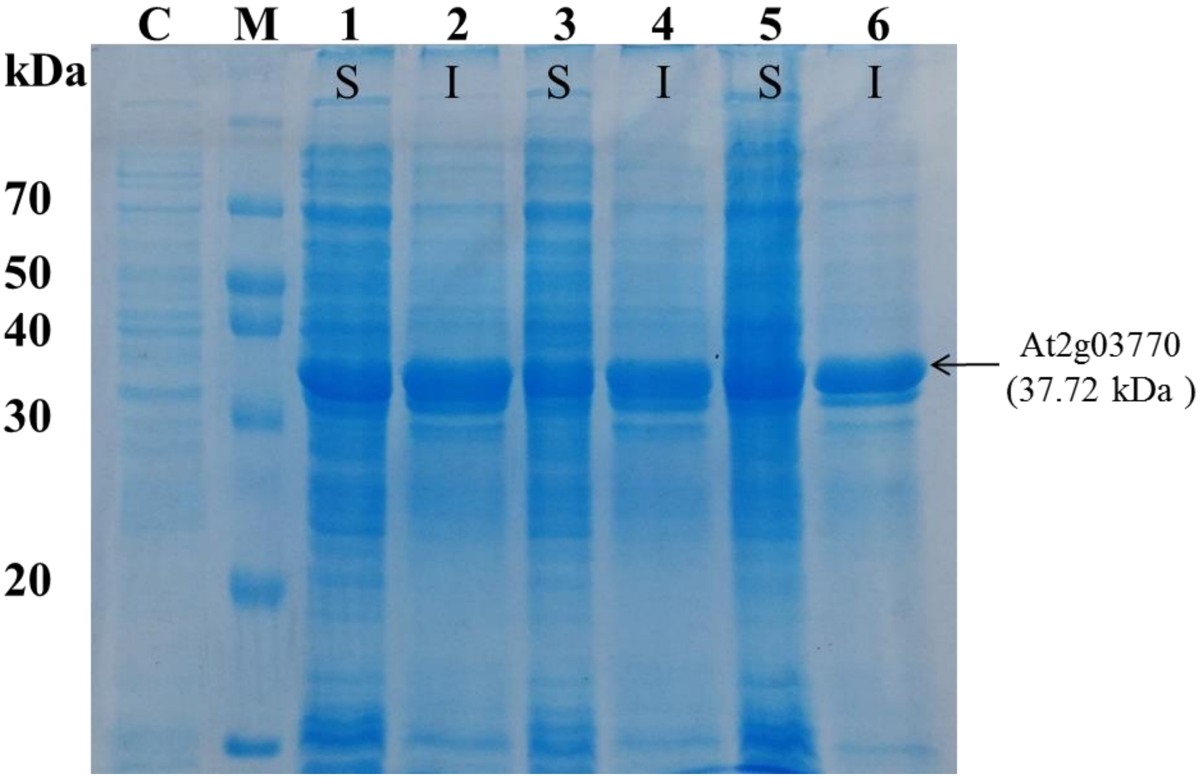

Figure S2. SDS-PAGE analysis of sulfotransferase At2g03770 (37.72 kDa) from *A.thaliana* expressed in BL21 (DE3). *Lane C*, control sample with out IPTG induction; *Lane M*, standard protein molecular mass maker; *Lane 1* and *Lane 2*, induced with 0.1 mM IPTG; *Lane 3* and *Lane 4*, induced with 0.5 mM IPTG; *Lane 5* and *Lane 6*, induced with 1.0 mM IPTG; S, soluble protein fraction; I, insoluble protein fraction.

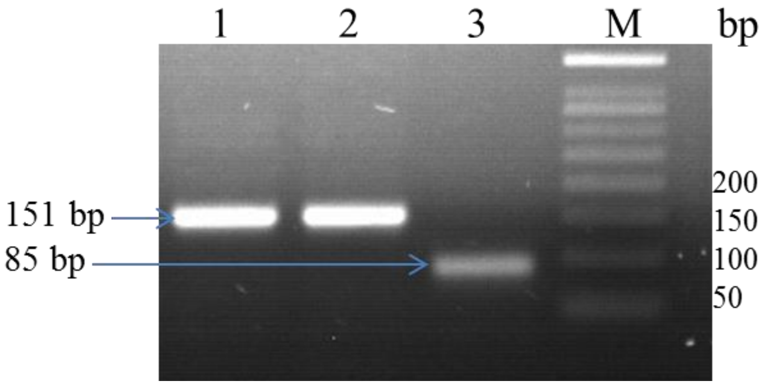

Figure S3. 2% agarose gel analysis. PCR results of colonies harboring CRISPRi-1 (*lane 1*), CRISPRi-2 (*lane 2*) and CRISPRi system (*land 3*) with 50bp DNA ladder marker (*lane M*). The synthetic gRNA obtained from positive clones of CRISPathBrick is 85 bp, while a 66 bp increase from colony PCR of CRISPRi-1 and CRISPRi-2.

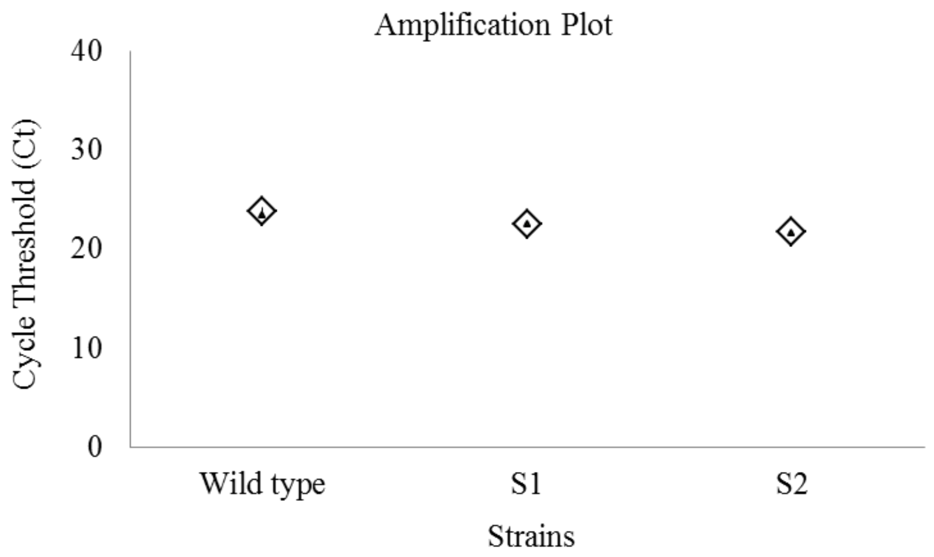

Figure S4. qRT-PCR cycle threshold values for 16S rRNA housekeeping gene expressed.

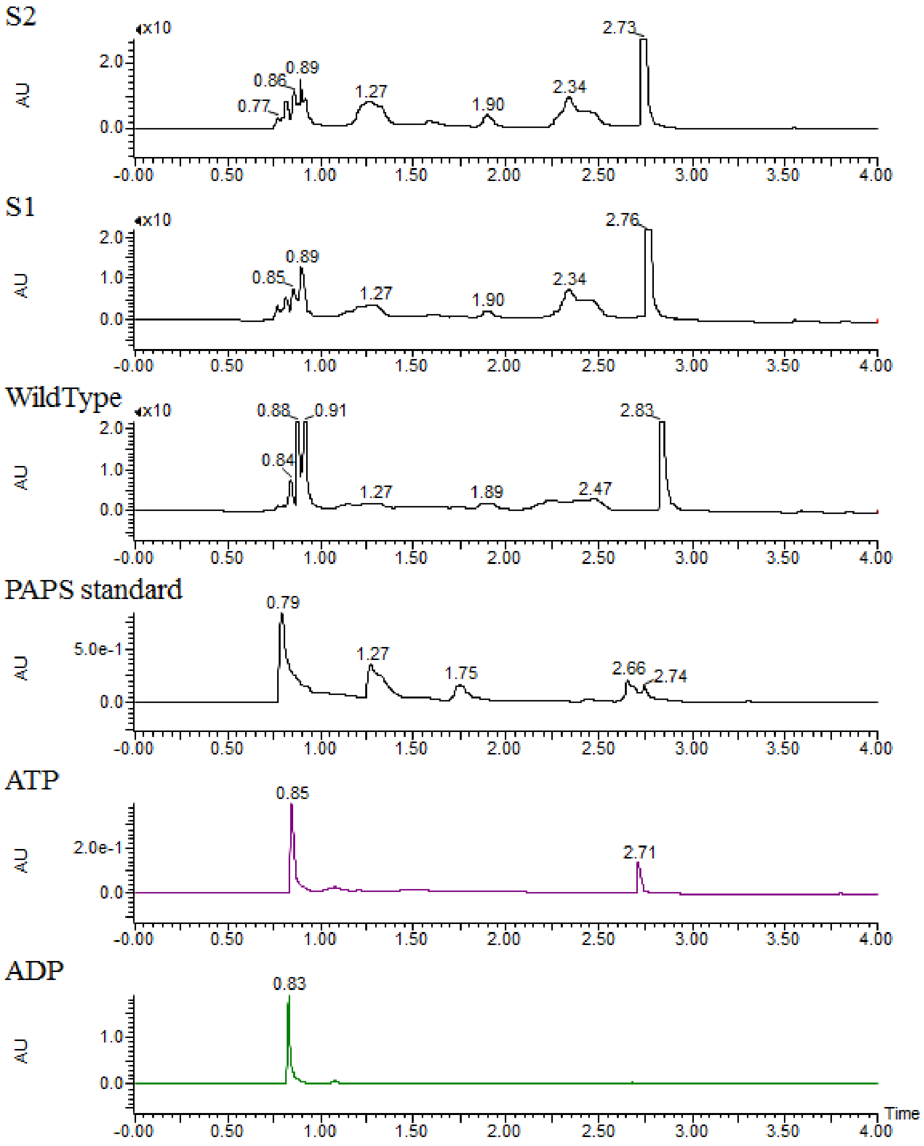

Figure S5. UHPLC-PDA coupled HR-QTOF ESI/MS under UV absorbance 254 nm of ADP, ATP, PAPS standard and PAPS obtained from three *E.coli* strains.

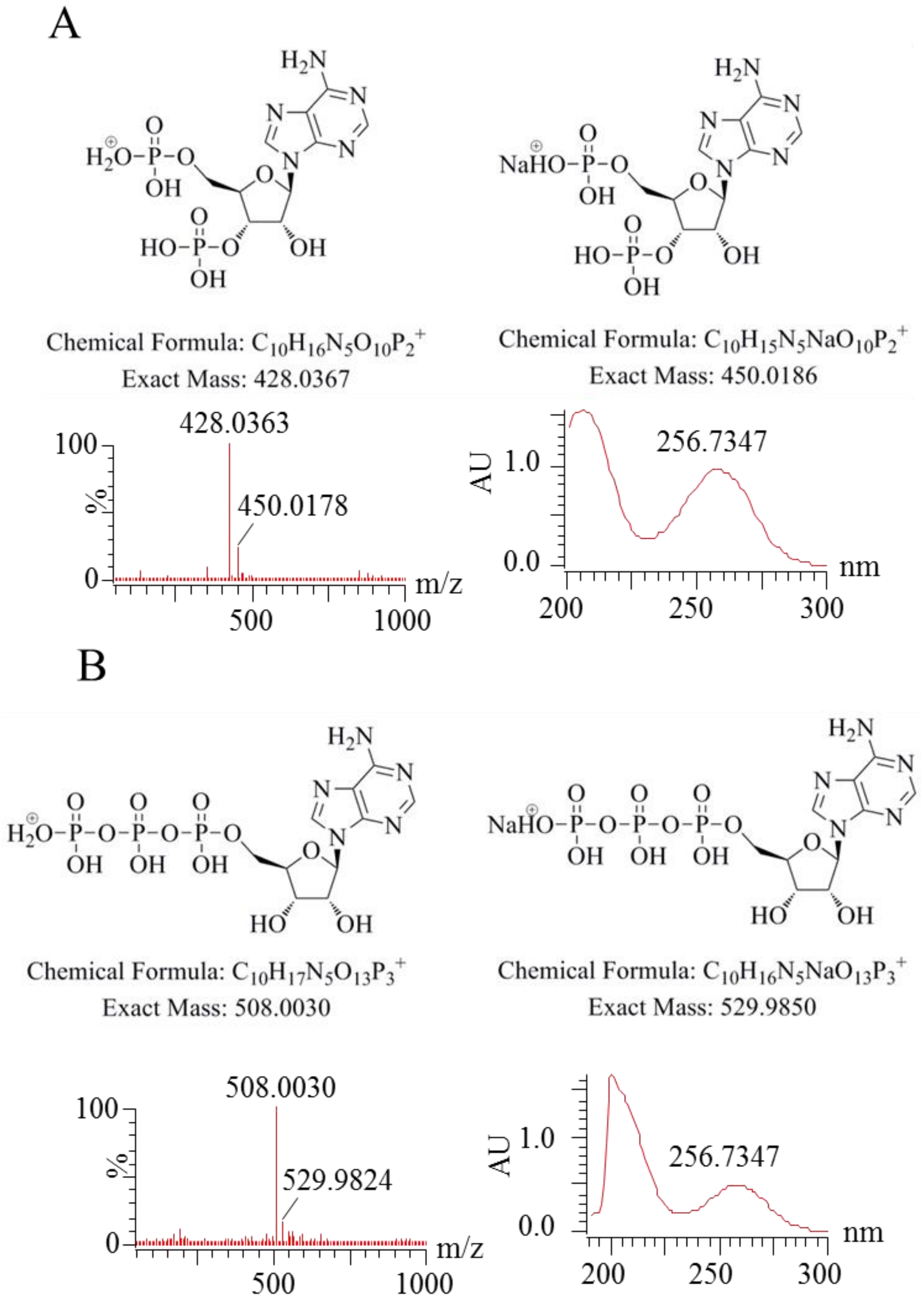

Figure S6. HR-QTOF ESI/MS analysis m/z$^+$ $[M+H]^+$ or $[M+Na]^+$ and the UV maxima absorbance of ADP (A) and ATP (B).

A) Exact mass of PAPS and fragmentation patterns

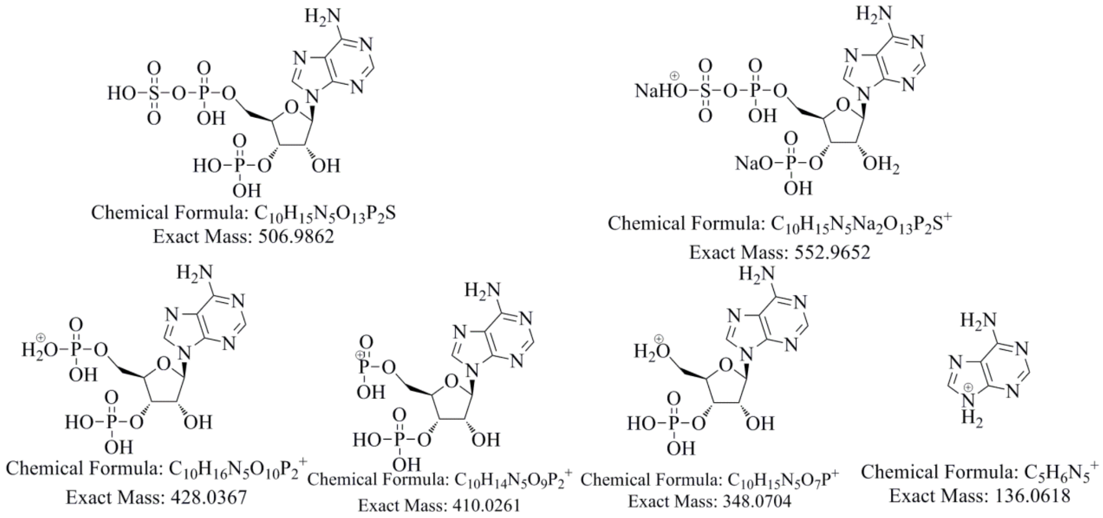

B) PAPS standard in positive ion mode ESI$^+$

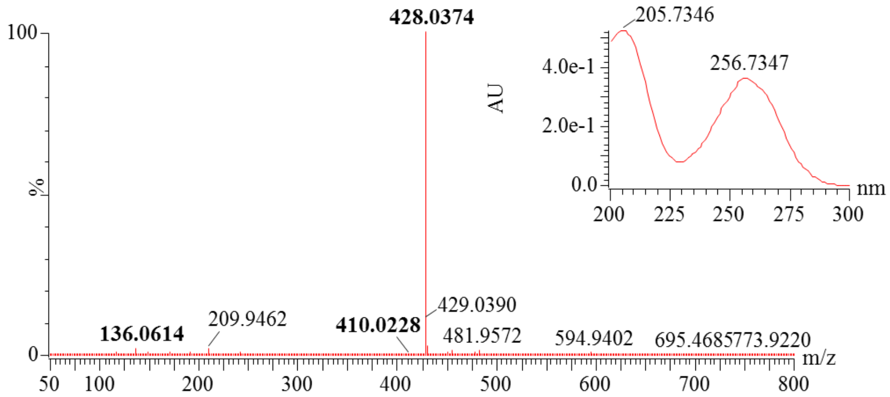

C) PAPS cytosol in positive ion mode ESI$^+$

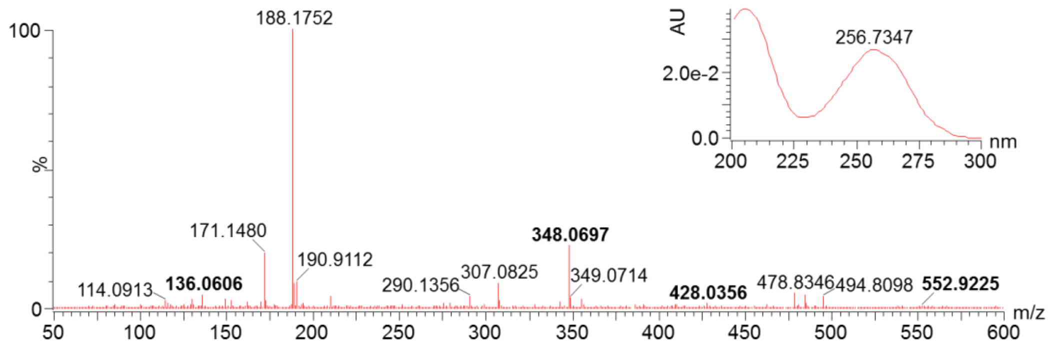

Figure S7. Fragmentation patterns, HR-QTOF ESI/MS and the UV maxima absorbance of PAPS standard and PAPS cytosol.

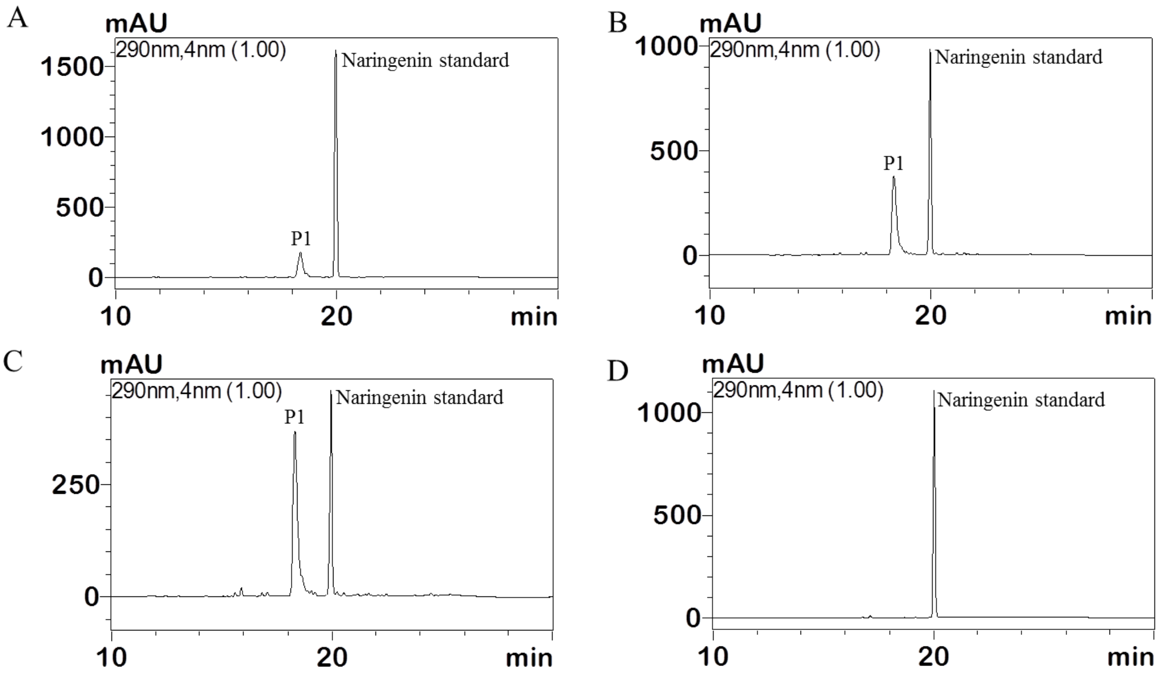

Figure S8. HPLC-PDA analysis of whole cells bioconversion of naringenin (t_R ~ 19.979) in three recombinant strains. (A) wild type, (B) S1 and (C) S2. (D) control reaction of substrate using *E.coli* BL21 (DE3). P1 has been identified as mono-sulfated naringenin (t_R ~ 18.248).

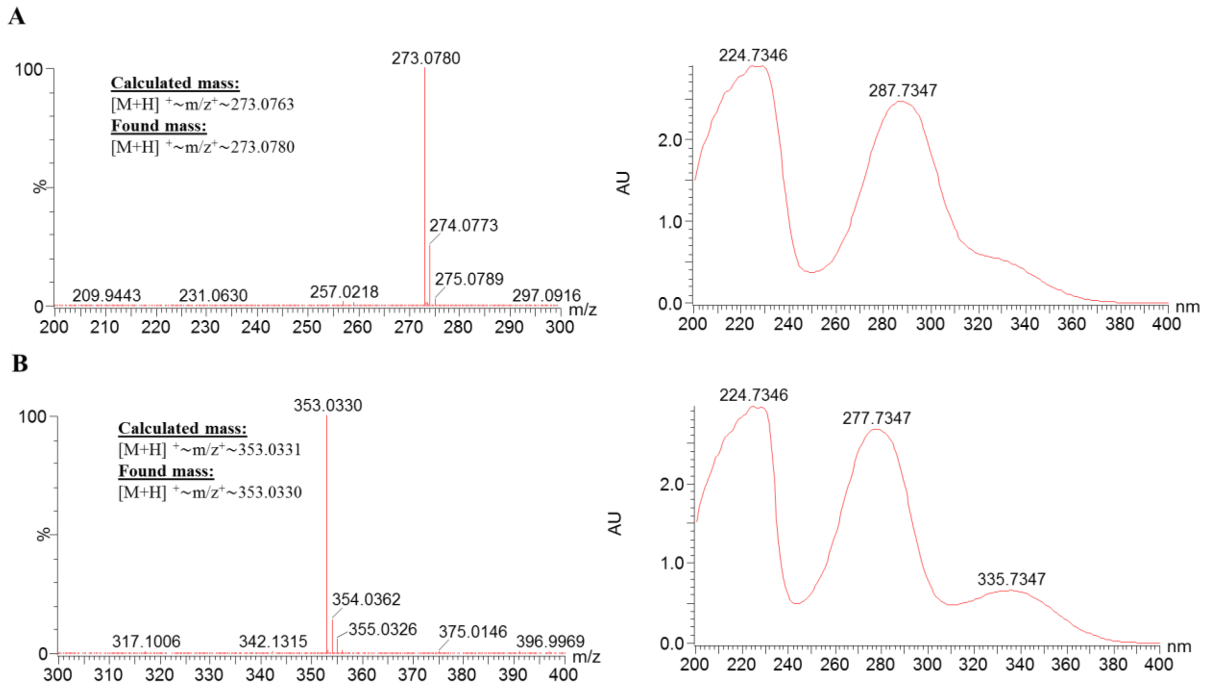

Figure S9. HR-QTOF ESI/MS and maxima UV absorbance of (A) naringenin standard and (B) reaction product.

A. ^{1}H-NMR of Naringenin standard

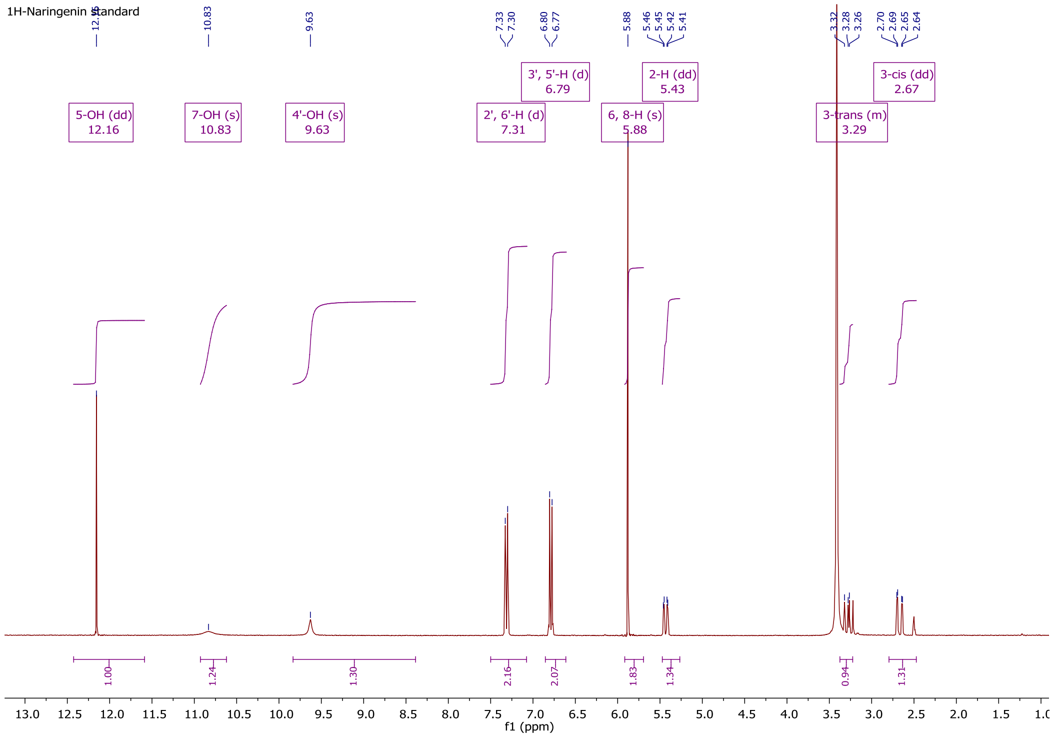

B. 1H-NMR of sulfated naringenin

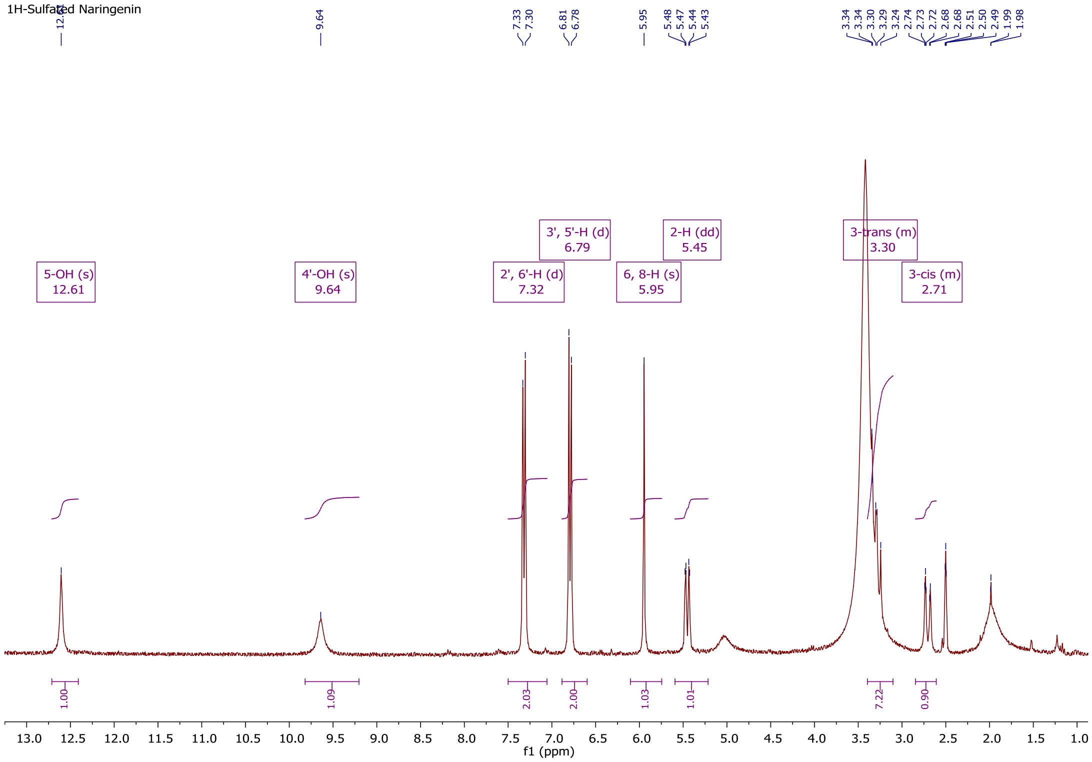

Figure S10. 1-Demensional NRM of Naringenin and its derivative

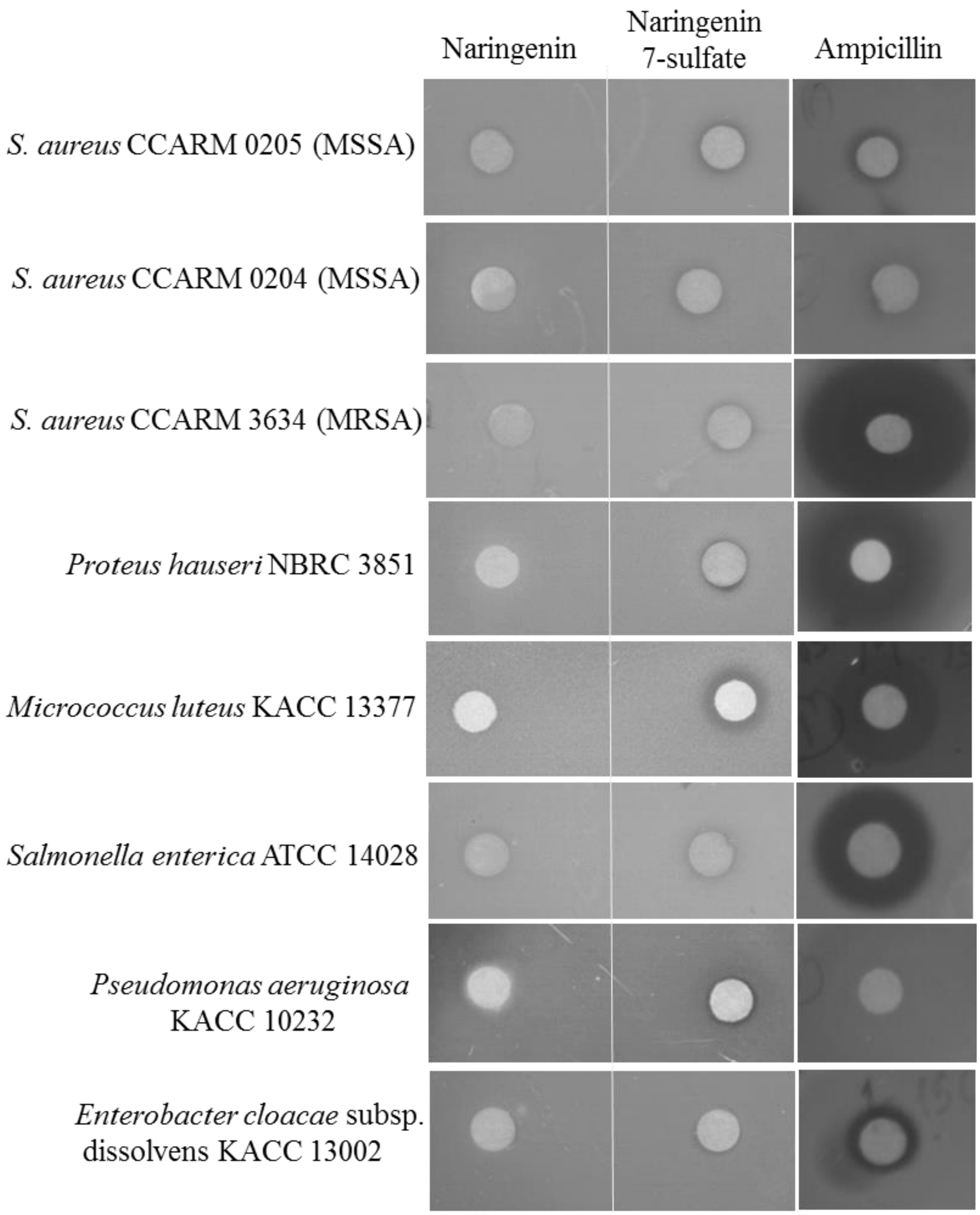

Figure S11. Antimicrobial assay plate for the antibacterial activity of naringenin and naringenin 7-sulfate against various Gram-positive and Gram-negative bacteria
